# MTO1-Deficient Mouse Model Mirrors the Human Phenotype Showing Complex I Defect and Cardiomyopathy

**DOI:** 10.1371/journal.pone.0114918

**Published:** 2014-12-15

**Authors:** Lore Becker, Eva Kling, Evelyn Schiller, Ramona Zeh, Anja Schrewe, Sabine M. Hölter, Ilona Mossbrugger, Julia Calzada-Wack, Valentina Strecker, Ilka Wittig, Iulia Dumitru, Tina Wenz, Andreas Bender, Michaela Aichler, Dirk Janik, Frauke Neff, Axel Walch, Leticia Quintanilla-Fend, Thomas Floss, Raffi Bekeredjian, Valérie Gailus-Durner, Helmut Fuchs, Wolfgang Wurst, Thomas Meitinger, Holger Prokisch, Martin Hrabě de Angelis, Thomas Klopstock

**Affiliations:** 1 Department of Neurology, Friedrich-Baur-Institute, Ludwig-Maximilians-University, Munich, Germany; 2 German Mouse Clinic, Helmholtz Zentrum München, German Research Center for Environment and Health, Neuherberg, Germany; 3 Institute of Experimental Genetics, Helmholtz Zentrum München, German Research Center for Environment and Health, Neuherberg, Germany; 4 Department of Cardiology, University of Heidelberg, Heidelberg, Germany; 5 Institute of Developmental Genetics, Helmholtz Zentrum München, German Research Center for Environment and Health, Neuherberg, Germany; 6 Institute of Pathology, Helmholtz Zentrum München, German Research Center for Environment and Health, Neuherberg, Germany; 7 Functional Proteomics, Goethe-University Frankfurt, Frankfurt am Main, Germany; 8 Institute for Genetics, University of Cologne, Cologne, Germany; 9 Research Unit Analytical Pathology – Institute of Pathology, Helmholtz Zentrum München, German Research Center for Environment and Health, Neuherberg, Germany; 10 Technical University Munich, Chair of Developmental Genetics, c/o Helmholtz Zentrum München, Neuherberg, Germany; 11 German Center for Neurodegenerative Diseases (DZNE), Munich, Germany; 12 Max-Planck-Institute of Psychiatry, Munich, Germany; 13 German Center for Vertigo and Balance Disorders, Munich, Germany; 14 Institute of Human Genetics, Helmholtz Zentrum München, German Research Center for Environment and Health, Neuherberg, Germany; 15 Deutsches Forschungszentrum für Herz-Kreislauferkrankungen (DZHK), partner site Munich Heart Alliance, Munich, Germany; 16 Institute of Human Genetics, Technical University Munich, Munich, Germany; 17 Chair of Experimental Genetics, Center of Life and Food Sciences Weihenstephan, Technical University Munich, Freising-Weihenstephan, Germany; 18 German Center for Diabetes Research (DZD), Neuherberg, Germany; 19 German Network for Mitochondrial Disorders (mitoNET), Munich, Germany; Virginia Commonwealth University, United States of America

## Abstract

Recently, mutations in the mitochondrial translation optimization factor 1 gene (*MTO1*) were identified as causative in children with hypertrophic cardiomyopathy, lactic acidosis and respiratory chain defect. Here, we describe an MTO1-deficient mouse model generated by gene trap mutagenesis that mirrors the human phenotype remarkably well. As in patients, the most prominent signs and symptoms were cardiovascular and included bradycardia and cardiomyopathy. In addition, the mutant mice showed a marked worsening of arrhythmias during induction and reversal of anaesthesia. The detailed morphological and biochemical workup of murine hearts indicated that the myocardial damage was due to complex I deficiency and mitochondrial dysfunction. In contrast, neurological examination was largely normal in *Mto1*-deficient mice. A translational consequence of this mouse model may be to caution against anaesthesia-related cardiac arrhythmias which may be fatal in patients.

## Introduction

Mitochondria are composed of more than 1500 proteins [1], almost all being encoded by nuclear DNA. In addition, mitochondrial DNA (mtDNA), a circular DNA with numerous copies in the mitochondrial matrix, encodes 22 transfer-RNAs (tRNAs), 2 ribosomal RNAs (rRNAs), and 13 polypeptides that are essential for electron transport and oxidative phosphorylation [2]. Mutations in mtDNA or nuclear-encoded mitochondrial genes lead to a plethora of metabolic, cardiovascular and neurodegenerative diseases and are a main culprit in aging [2], [3], [4]. Nuclear mutations may reside in genes coding for respiratory chain subunits or their assembly factors or proteins necessary for replication, transcription or translation of mtDNA [5], [6], [7], [8].

Defects in mitochondrial translation are increasingly recognized as a cause of human diseases. The intricate machinery of mitochondrial protein synthesis may be disturbed at any step, including mutations in mtDNA-encoded tRNAs and rRNAs as well as mutations in nuclear-encoded aminoacyl-tRNA synthetases, ribosomal proteins, elongation factors, termination factors, mRNA stability factors, and translation activators [9]. Moreover, four distinct defects have been described so far in tRNA-modifying enzymes. tRNA modification is critical for folding and codon recognition. Modifications of the first base of the anticodon, the so-called wobble position for example, enable tRNAs to recognize multiple codons.

Mutations in the pseudouridine synthase I (PUS1) gene hamper the process of pseudouridylation, and lead to the rare recessive syndrome of mitochondrial myopathy, lactate acidosis and sideroblastic anemia (MLASA) (MIM 600462) [10]. Mutations of methionyl-tRNA formyltransferase (MTFMT) have been described in four patients presenting with Leigh syndrome and isolated complex I or combined oxidative phosphorylation (OXPHOS) deficiency [11], [12]. The mitochondrial translation optimization factor 1 (MTO1; MGI:1915541) as well as the tRNA 5-methylaminomethyl-2-thiouridylate methyltransferase (TRMU = mitochondrial translation optimization factor 2, MTO2) are described to be responsible for the highly conserved 5-carboxymethylaminomethylation (mnm5s2U34) modification of the wobble uridine base in glutamine, glutamate, and lysine tRNAs in bacteria, and yeast thus contributing to the optimization of mtDNA-dependent protein synthesis [9], [13], [14]. Mutations of *TRMU* in humans cause acute liver failure, benign COX deficiency or Mitochondrial Infantile Liver Disease [15], [16], [17], [18]. Recently, the first human mutations in *MTO1* were identified by exome sequencing in two siblings and a third unrelated individual with infantile hypertrophic cardiomyopathy, lactic acidosis and a respiratory chain defect [19] (MIM 614667). Another study reported *MTO1* mutations in five additional patients with either complex IV or combined complex I and IV deficiency. In addition to hypertrophic cardiomyopathy and lactic acidosis, some patients also displayed neurological signs: out of the eight patients described, four had muscle hypotonia, two showed psychomotor delay;, one had a moderate bilateral optic atrophy and one suffered from encephalopathy and seizures [20]. Four patients have already died, two from sudden bradycardia, one from cardio-respiratory arrest, and one from unknown reasons [20]. So far, no mouse model for this human condition was available.

The *Mto1* gene is located on chromosome 9 in the mouse genome and on chromosome 6 in humans. The mouse *Mto1* gene contains 12 exons and encodes a 669 residues protein exhibiting strong similarity (87%) with its human homolog. *Mto1* is ubiquitously expressed in various tissues, but with a markedly elevated expression in tissues with high metabolic rates [21].

The aim of this study was to establish a mouse model for human MTO1 deficiency, and to comprehensively investigate its phenotype and biochemistry.

## Results

### Generation of *Mto1* knockdown mice by gene trap mutagenesis

**Figure 1:**
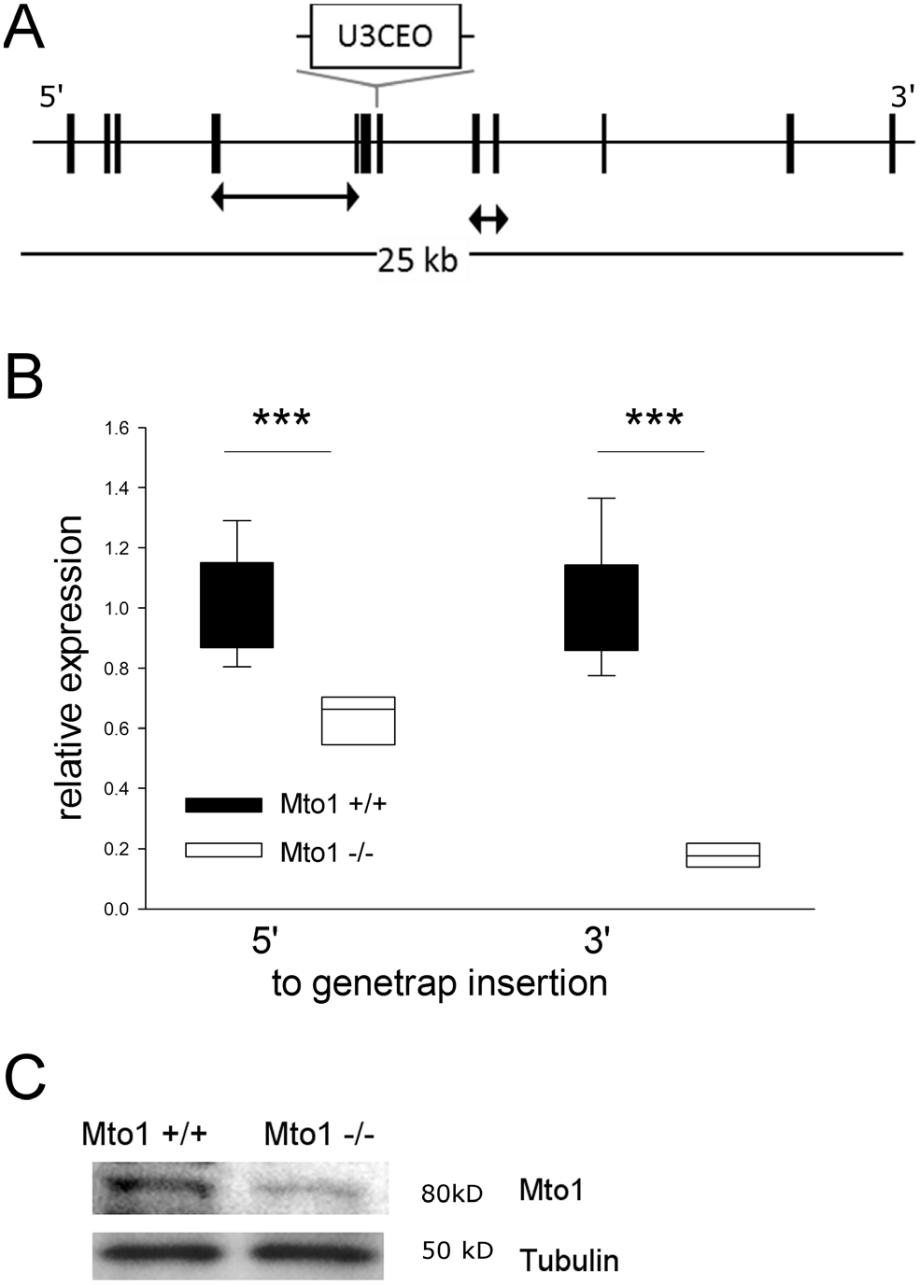
Generation of *Mto1* knockdown mice by gene trap mutagenesis. (A) Integration of gene trap vector U3CEO in intron 6 of *Mto1*. Arrows indicate amplificons for RT-PCR. (B) RT-PCR of heart tissue shows reduced levels of *Mto1* transcripts using primers covering sequences 5′ or 3′ to the integration site, ***p<0.001. (C) Western blot showing a clear reduction of MTO1 protein in *Mto1*−/− mouse embryonic fibroblasts as compared to *Mto1*+/+ controls.

Mutant mice were generated by insertion of the gene trap vector U3CEO into intron 6 of the *Mto1* gene by the German Gene Trap Consortium (Mto1^Gt(G019A03)Wrst^); Fig. 1A). The *Mto1* specific integration was identified by sequencing of the fusion transcript and confirmed by DNA sequencing (data not shown). Quantitative RT-PCR analysis of *Mto1* mRNA expression levels in heart from homozygous *Mto1* mutant mice (n = 9, age 10 weeks) with primers covering a region 5’ to the integration site of the gene trap vector (thus detecting mutated and non-mutated transcripts) revealed a significant reduction of *Mto1* transcripts (63%) compared to controls (t-test, p<0.001). The analysis of full-length transcripts revealed 17% residual transcripts compared to wild-type (Fig. 1B). Hence, the gene trap created a hypomorphic *Mto1* allele.

Two commercially available antibodies were not successful in MTO1 protein detection by Western blots of mouse tissue preparations. One of these antibodies did, however, work on isolated mouse embryonic fibroblasts showing a clear reduction of MTO1 in mutants as compared to controls (Fig. 1C).

Mice were bred on a mixed genetic background (B6129 S/J). No abnormalities were observed in heterozygous offspring and their breeding gave birth to homozygous mutants in a Mendelian ratio. Fertility, breeding as well as offspring ratios were unaffected in homozygous and heterozygous mutants. Lifespan was not affected under normal conditions and mutants also reached high ages (Figure S1 in S1 File).

### Phenotypic analysis of *Mto1* mice revealed cardiological alterations

Comprehensive phenotypic analysis was performed in the German Mouse Clinic [22], [23] with a cohort of 60 young adult mice (15 per sex and genotype) in a primary screening pipeline. Phenotyping started at the age of 8 weeks and lasted 14 weeks; accordingly, mice from this first analysis underwent pathological examination at the age of 22 weeks. This primary screen included analyses from 14 medical disciplines including neurology, behavior, nociception, vision, cardiovascular function, energy metabolism, immunology, dysmorphology, clinical chemistry, and molecular phenotyping. We will focus here on the results of the neurological, behavioral, and cardiovascular analysis, since those were the screens of major interest for mitochondrial disorders. In addition to the primary analyses, we performed further tests in additional cohorts and at different ages.

No obvious changes between mutant and wild-type mice were found in basic observation tested according to a modified SHIRPA protocol. Body weight at the age of 10 weeks was significantly reduced (male mutants 25.7±0.5 g versus control 28.4±0.6 g; female mutants 22.1±0.8 g versus control 24.5±0.9 g; p<0.001). Grip strength measurement showed a tendency towards more strength in mutants. The lighter *Mto1* mutant females showed a trend to improved performance on an accelerating rotarod whereas mutant males showed impaired performance in advancing trials (data not shown). Replication of neurological analysis in a cohort of aged mice (16–20 months old) showed again no significant differences in SHIRPA, grip strength and rotarod performance between mutants and wild-type (data not shown).

**Figure 2:**
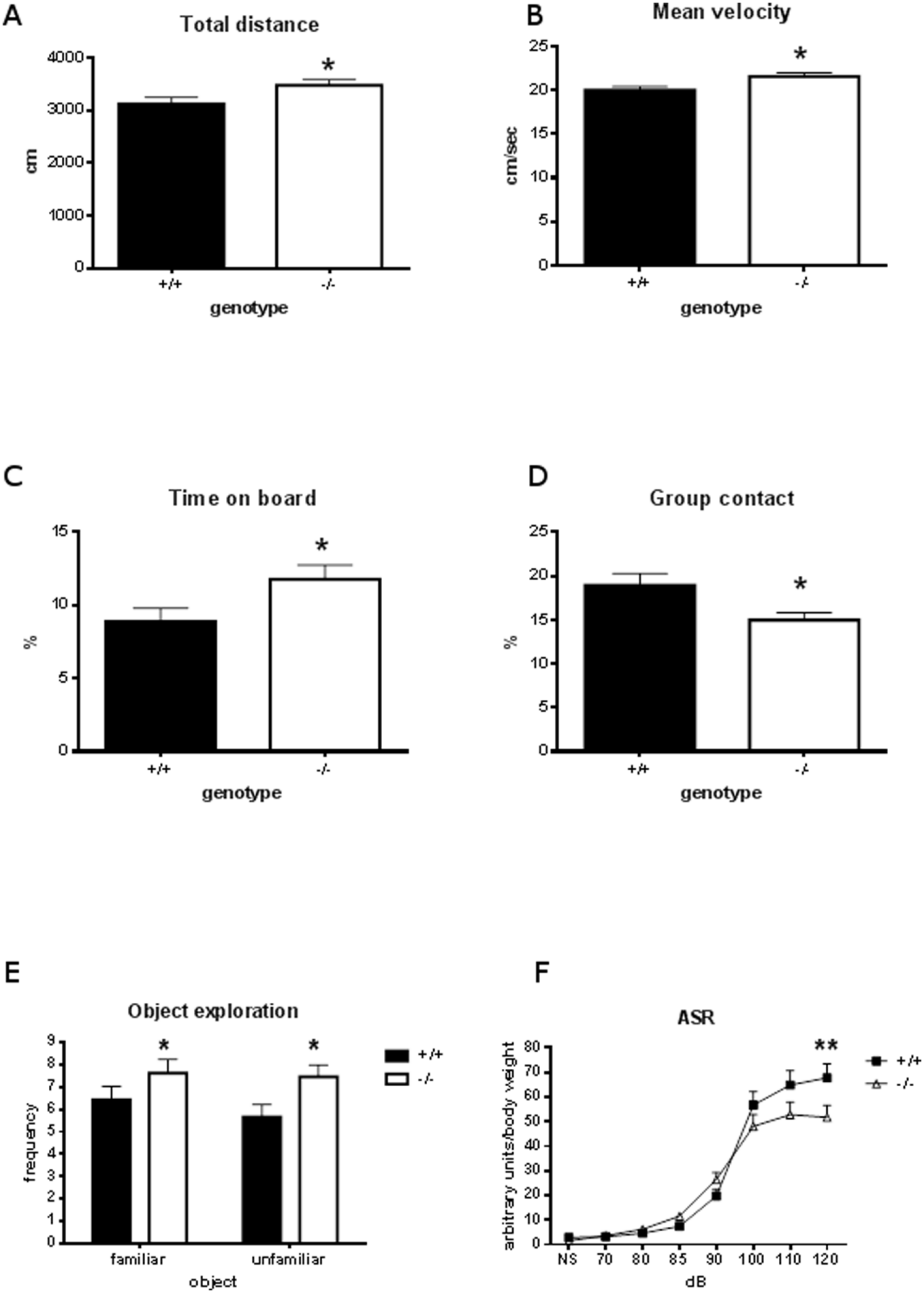
Behavioral analysis of *Mto1* mutant mice using a modified holeboard. *Mto1*−/−mice show increased horizontal locomotor activity (A) as well as increased mean velocity (B) as compared to controls. Anxiety-related behavior measured via the time on board (C) and group contact (D) was also significantly changed; object exploration was increased (E) and acoustic startle response decreased (F), *p<0.05; **p<0.01.

When tested for spontaneous behavior in a novel environment in the modified Hole Board test, *Mto1* mice (aged 8 weeks) of both sexes exhibited increased horizontal locomotor activity as indicated by total distance travelled, mean velocity and object exploration, as well as reduced anxiety-related behavior as measured by increased percentage of time spent on the board in the anxiogenic centre of the test arena and reduced percentage of time spent at the partition, i.e. in group contact with the cage mates (Figs. 2A–E). Acoustic startle response measured in another cohort of mice (9 controls and 10 mutants aged 9 weeks) showed an altered curve: there was a highly significant interaction between genotype and startle pulse intensity (F_(7,11)_ = 4.153, p<0.001), and Bonferroni posthoc tests revealed a significantly lower response of mutants at maximum dB (p<0.01, Fig. 2F).

**Figure 3:**
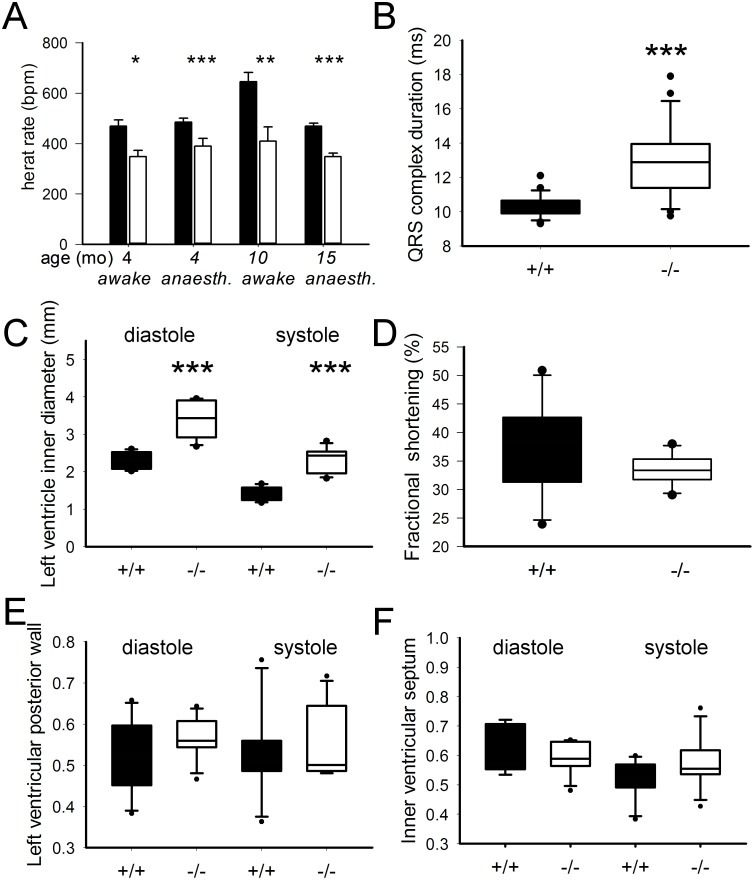
Cardiovascular analysis. (A) Heart rate was reduced in *Mto1*−/− mutants (white bars) as compared to controls (black bars) at different ages and under different conditions in three cohorts of mice (4, 10 and 15 months old). (B). Electrocardiography in anaesthetized mice showing increased QRS complex duration in *Mto1*−/− mutant mice as compared to controls (C−F) Echocardiography in awake mice showing increased left ventricular internal diameter in systole and diastole in *Mto1*−/− mutant mice (C) but no significant differences in fractional shortening (D), left ventricular posterior wall thickness (E) and intraventricular septum thickness (F), *p<0.05; ***p<0.001.

Heart rate was consistently reduced in mutants as compared to controls at different ages and conditions: (i) awake mice at age 4 months (males: mutant 521.3+/−30.1 bpm versus control 566.9+/−26.2 bpm; females: mutant 518.2+/−19.3 bpm versus controls 590.3+/−22.5 bpm, ANOVA genotype effect p<0.05); (ii) anaesthetized mice at age 4 months (males: mutant 392.8+/−29.3 bpm versus control 486.1+/−16.9 bpm; females: mutant 387.6+/−31.8 bpm versus controls 483.7+/−14.2 bpm, ANOVA genotype effect p<0.001), Table 1); (iii) awake mice at age 10 months (mutant 409+/−57.4 bpm versus control 645+/−36.5 bpm; p<0.01); (iv) anaesthetized mice at age 15 months (males: mutant 357.6+/−14.6 bpm versus control 466.1+/−10.7 bpm; females: mutant 339.1+/−13.7 bpm versus controls 472.1+/−12.7 bpm, ANOVA genotype effect p<0.001; Fig. 3A).

**Table 1:** ECG parameters (Data are presented as mean +/− standard error of mean).

	Males	MALES	Females	FEMALES			
	Mto1+/+	Mto −/−	Mto1+/+	Mto−/−	Sex	Genotype	Interact.
**N = **	**10**	**10**	**10**	**9**	**p - value**	**p - value**	**p - value**
**PQ interval [ms]**	37.5+/−1.2	36.4+/−1.3	40.3+/−0.9	41.6+/−2.9	**p<0.05**	**n.s.**	**n.s.**
**P-wave duration [ms]**	19.2+/−0.5	19.9+/−0.6	20+/−0.3	19.7+/−0.5	**n.s.**	**n.s.**	**n.s.**
**QRS-complex duration [ms]**	10.4+/−0.2	13.1+/−0.8	10.2+/−0.2	13+/−0.6	**n.s.**	**p<0.001**	**n.s.**
**QT interval [ms]**	43.1+/−0.8	51.6+/−2.8	43.3+/−0.9	48.4+/−3.4	**n.s.**	**p<0.01**	**n.s.**
**QT_corrected_ [ms]**	38.6+/−0.6	40.8+/−1.0	38.7+/−0.4	37.9+/−1.6	**n.s.**	**n.s.**	**n.s.**
**RR interval [ms]**	125.2+/−4.6	160.3+/−11	125.4+/−3.8	165.2+/−15.8	**n.s.**	**p<0.001**	**n.s.**
**Heart rate [bpm]**	486.1+/−16.9	392.8+/−29.3	483.7+/−14.2	387.6+/−31.8	**n.s.**	**p<0.001**	**n.s.**
**JT interval [ms]**	3.1+/−0.1	3.1+/−0.1	3.3+/−0.1	4.3+/−0.7	**n.s.**	**n.s.**	**n.s.**
**ST interval [ms]**	32.7+/−0.8	38.5+/−2.4	33+/−0.9	35.4+/−3	**n.s.**	**p<0.05**	**n.s.**
**Q amplitude [mV]**	0.01+/−0	0.01+/−0	0.02+/−0	0.01+/−0	**n.s.**	**n.s.**	**n.s.**
**R amplitude [mV]**	3.25+/−0.18	3.25+/−0.35	3.7+/−0.25	3.11+/−0.19	**n.s.**	**n.s.**	**n.s.**
**S amplitude [mV]**	−0.66+/−0.08	−0.39+/−0.27	−0.93+/−0.16	−0.33+/−0.07	**n.s.**	**p<0.05**	**n.s.**
**QRS amplitude [mV]**	3.9+/−0.22	3.66+/−0.53	4.63+/−0.32	3.46+/−0.18	**n.s.**	**p<0.05**	**n.s.**
**Arrhythmias**	**[# of animals]**						
** - SVES**	0	0	1	0			
** - VES**	0	3	0	4			
** - AV block**	0	5	0	6			
** - SA block**	**0**	**3**	**0**	**4**			
** - others**	0	8	0	8			
**Regular ECG**	10	2	9	1		**p<0.001**	**Fischer exact**

In electrocardiography (ECG) of anaesthetized mice, QRS complex duration was markedly increased (Fig. 3B). Q-T interval was slightly but significantly increased and QRS amplitude was significantly decreased in mutants (Table 1). Qualitative analysis of the ECG demonstrated an extremely high incidence of arrhythmic events in mutant animals (Table 1). While only one out of 20 control animals had premature atrial beats, 16 of 19 mutant animals had marked arrhythmias (p<0.001), mainly atrioventricular blocks, but also premature ventricular beats or a higher degree of sinoatrial node blocks seen as an intermittent complete loss of the P wave. These marked arrhythmias occurred especially during induction and reversal of anaesthesia. ECG in an additional cohort of older mice (15 months old, Table S1 in S1 File) confirmed these altered parameters. ECG without anaesthesia was performed in five mice, and no arrhythmias were encountered here.

**Figure 4:**
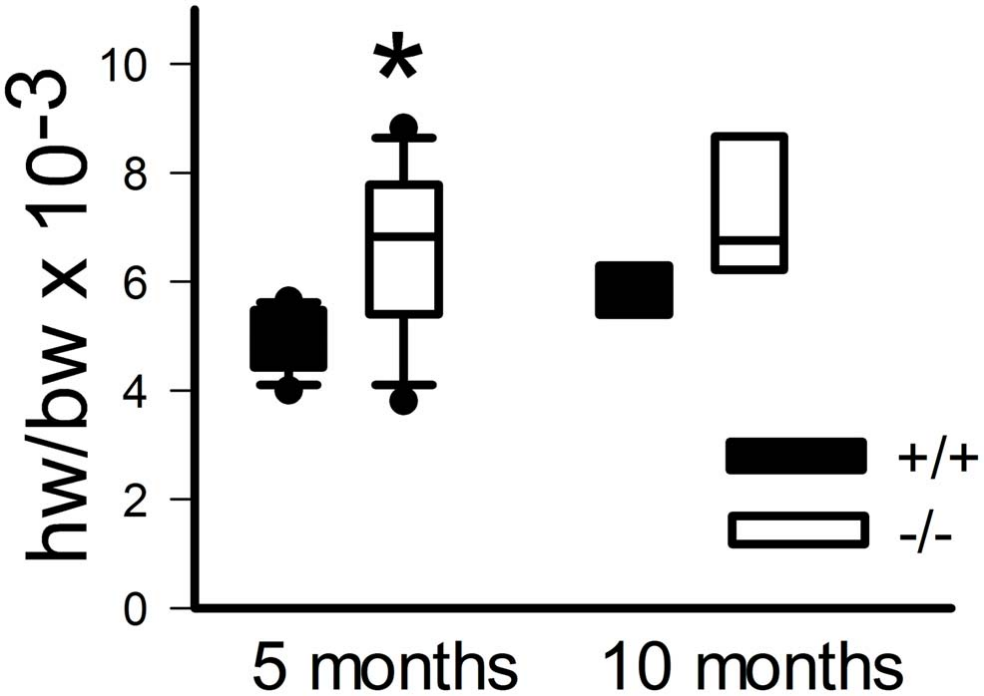
Heart weight related to body weight was significantly increased in *Mto1*−/− mutant mice as compared to controls, both in 5 months old mice (n = 15, *p<0.05) and as a trend in 10 months old mice (n = 8, n.s.).

At echocardiography, we detected slightly dilated hearts in the knockout mice with significantly increased diastolic and systolic internal LV diameters (p<0.001 each; Fig. 3C). However, LV function was preserved with insignificant differences in fractional shortening between both groups (+/+ n = 6; 37.65131+/−3.86948; −/− n = 7; 32.43615+/−1.49116; t-test n.s., Fig. 3D). Posterior wall and septum thickness did not show significant differences between both groups (Fig. 3E, F). Heart weight at the age of 22 weeks was significantly increased related to body weight in mutants compared to controls (mutant heart weight/body weight = 0.00654+/−0.00065; control hw/bw = 0.00490+/−0.00021; n = 15, p<0.05, Fig. 4). In 10 months old animals this was seen as a trend (mutant heart weight/body weight = 0.00745+/−0.00101; control hw/bw = 0.00585+/−0.00031; n = 8, n.s., Fig. 4).

The heart weight differences pointing towards cardiomyopathy were not reflected in the histopathological analysis of the young hearts. A total of 23 *Mto1* mutant mice (13 females, 10 males) and 23 control mice (14 females, 9 males) were analyzed macroscopically. Histological analysis was performed in 22 mice. Necropsy and histological analysis of 30 organs using HE staining showed no gross abnormalities in the mutants of both sexes analyzed at the end of the primary screen test battery at the age of 22 weeks although functional changes were already detected at this age.

**Figure 5:**
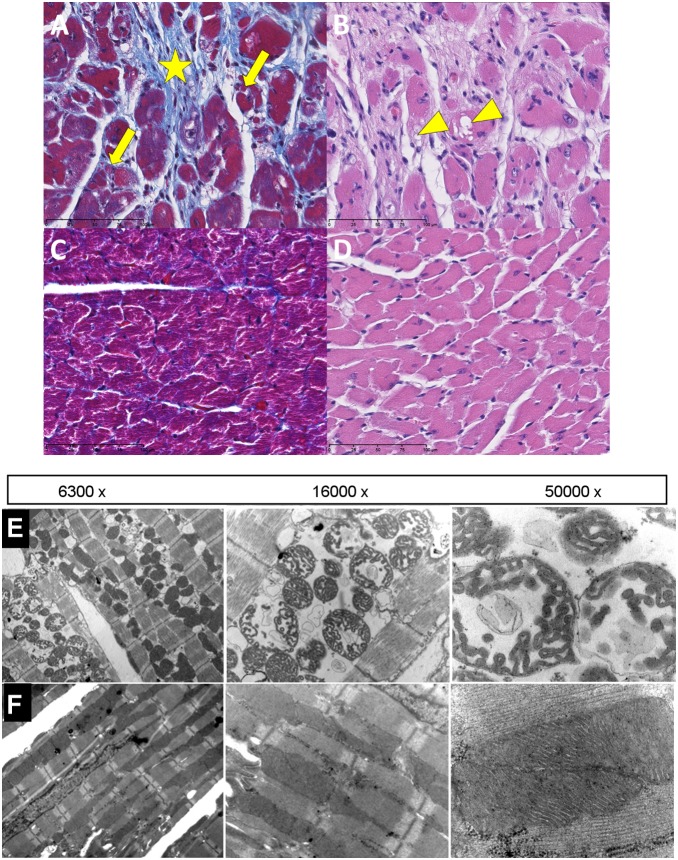
Morphological analysis of heart. (A, C) Focal myocard degeneration in the left ventricle with myofiber atrophy(arrows) and fibrosis(asterisk) in a 303 day old Mto1−/− mouse (A) as compared to control (C) (Masson stain, scale bar 200 µm). (B, D) Focal vacuolar degeneration (arrowheads) of myofibers in the heart of a 303 day old MTO1−/− mouse (B) as compared to control (D) (H&E stain, scale bar 200 µm). (E, F) Transmission electron microscopy showing degeneration of heart muscle in Mto1−/−mice (upper panel E) as compared to wild-type (lower panel F) at different magnifications (as indicated).

However, histopathological examination of hearts from the 10 months old mice revealed focal signs of myofiber degeneration (e.g. atrophy and vacuolization) and fibrosis in the mutant animals (Fig. 5A, B) as compared to wildtype controls (Fig. 5C, D). Additionally, unspecific age-related focal mineralization of the epicardium and myocardium were found in all mutant and control mice. Neither amyloid deposits (Congo red stain) in the heart nor hemosiderin-laden pulmonary alveolar macrophages (“heart failure cells”, Prussian blue staining) were found in mutants or controls. Analyzing hearts in more detail with Transmission Electron Microscopy revealed focal necrosis in cardiac muscle cells with intracristal swelling in mitochondria and dilated sarcoplasmic reticulum in young adult mutant mice (Fig. 5E) as compared to wildtype controls (Fig. 5F).

### MTO1 deficiency leads to reduced mtDNA copy number and OXPHOS impairment

**Figure 6:**
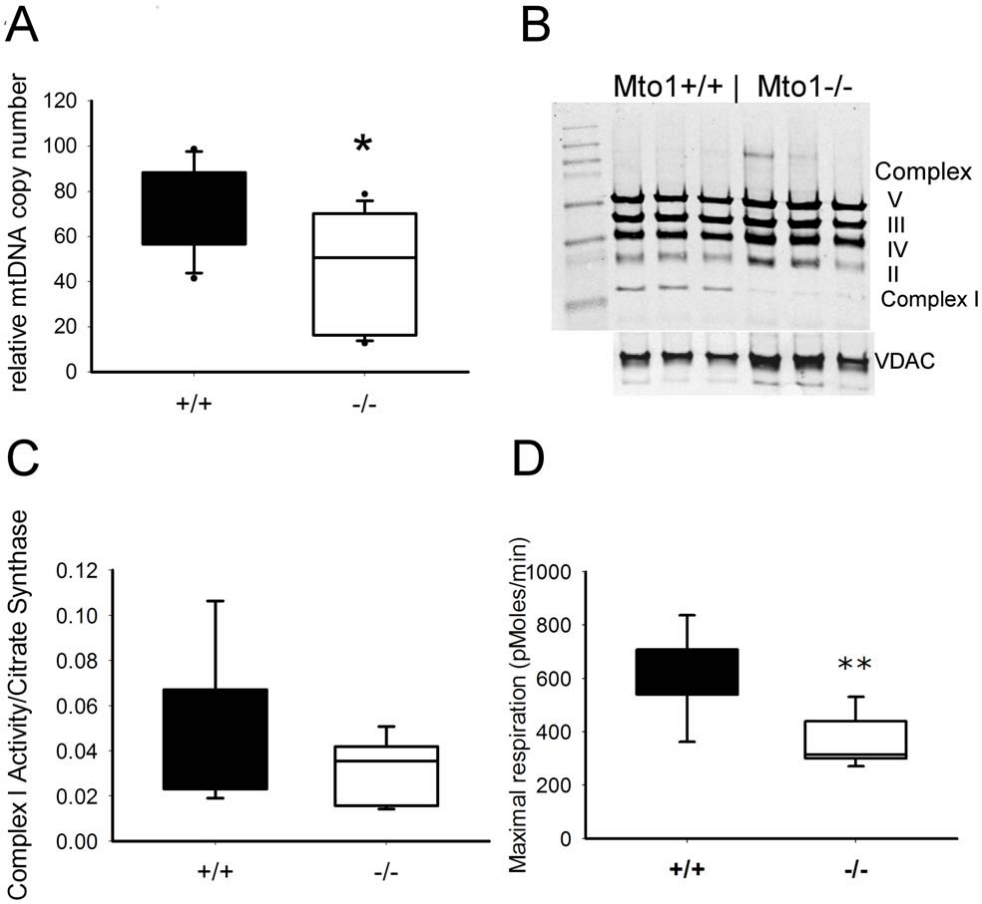
Molecular analysis of mouse tissue. (A) mtDNA copy number in hearts of *Mto1*−/− mutant mice was significantly reduced as compared to controls (*p<0.05) (B) Respiratory chain complex I was visibly reduced in a Western Blot using a cocktail of OXPHOS antibodies (Mitosciences.com) in *Mto1*−/− mutant mice as compared to controls while respiratory chain complexes II, II, IV and V were unchanged. (C) Enzymatic activity of complex I in heart tissue lysates was only mildly reduced in *Mto1*−/− mutant mice but (D) maximal respiratory capacity was significantly reduced in freshly isolated mitochondria (**p<0.01).

The relative copy number of heart mtDNA was determined in the same batch of mice used for expression analysis by calculating the mtDNA ND1 *(NADH dehydrogenase 1*) gene copies relative to the nuclear encoded house-keeping gene ACTB using real-time quantitative PCR [24]. The copy number was reduced to about 60% of that of controls (Fig. 6A, p = 0.018, n = 9).

Since altered translation in mitochondria is supposed to have a major effect on respiratory chain complex (RCC) proteins, the relative amount of RCC complexes was tested in a Western blot of heart mitochondria isolated from control and mutant mice and probed with an antibody cocktail against several subunits of RCC (Mitosciences). Notably lower amounts of complex I protein were detected in samples from mutant mice whereas there was no difference between mutants and control mice in the other complexes (Fig. 6B).

Analyzing the enzymatic activity of RCC complexes revealed a tendency towards reduced complex I activity in preparations of frozen hearts but this reduction did not reach significance (n = 5 each; Fig. 6C). No differences were detected in complex II/III and complex IV activity in heart and liver (data not shown). Oxygen consumption of freshly isolated heart mitochondria was measured with Seahorse technology. Maximal respiration rate (MRR) (or state 3_u_) of isolated heart mitochondria was calculated as the difference between FCCP-uncoupled maximal respiration rate and the respiration rate after electron flow from complex II was inhibited by the complex III inhibitors antimycin A and myxothiazol. Maximal respiration rate normalized to total protein was significantly reduced in mutant mitochondria (p = 0.008, Fig. 6D). The respiratory control ratio (RCR) defined as ratio state3/state4o with succinate as substrate in the presence of the complex I inhibitor rotenone was not significantly different (mutants 4.0+/−1.5; controls 3.5+/−0.9; p = 0.515). Citrate synthase activity as a marker for the amount of mitochondrial mass was not altered (data not shown).

**Figure 7:**
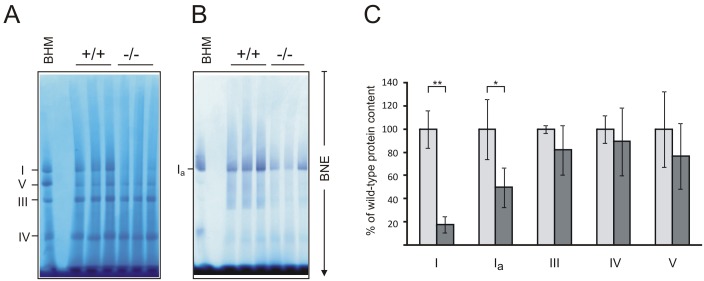
Blue Native Gel electrophoresis of heart tissue. Mitochondrial complexes from wild-type and *Mto1*−/− heart homogenates were solubilized with dodecyl-β-d-maltoside and separated by BNE. (A) Coomassie stained 1-D BNE gels, (B) 1-D BNE/complex I in gel activity stain were used for (C) densitometric quantification of mitochondrial complexes. Complex values from 3 wild-type and 3 *Mto1*−/− mice are expressed as percent of the wild-type mean. The Complex I defect is confirmed. Assignment of Coomassie stained complexes: I, complex I or NADH dehydrogenase, V, monomeric complex V or ATP synthase; III, complex III or cytochrome c reductase; IV, complex IV or cytochrome c oxidase; Ia, in-gel activity stain; *, significant differences (p<0.05); **, significant differences (p<0.01) by Students t test; error bars indicate standard deviation (SD).

A detailed analysis of OXPHOS complexes was carried out using blue native electrophoresis (BNE). Mitochondrial membranes from hearts were solubilized and OXPHOS complexes were isolated by BNE. Densitometric quantification of Coomassie stained protein complexes and *in-gel* complex I activity confirmed reduced abundance of complex I in preparations of mutant hearts compared to controls (Fig. 7). We did not detect any changes in the abundance of any complex in skeletal muscle, liver and brain. There was a tendency towards reduced complex I activity also in skeletal muscle but this was not statistically significant (Supporting Information, Figures S2–5 in S1 File).

### Knockdown of *Mto1* alters *de* *novo* mitochondrial protein synthesis

**Figure 8:**
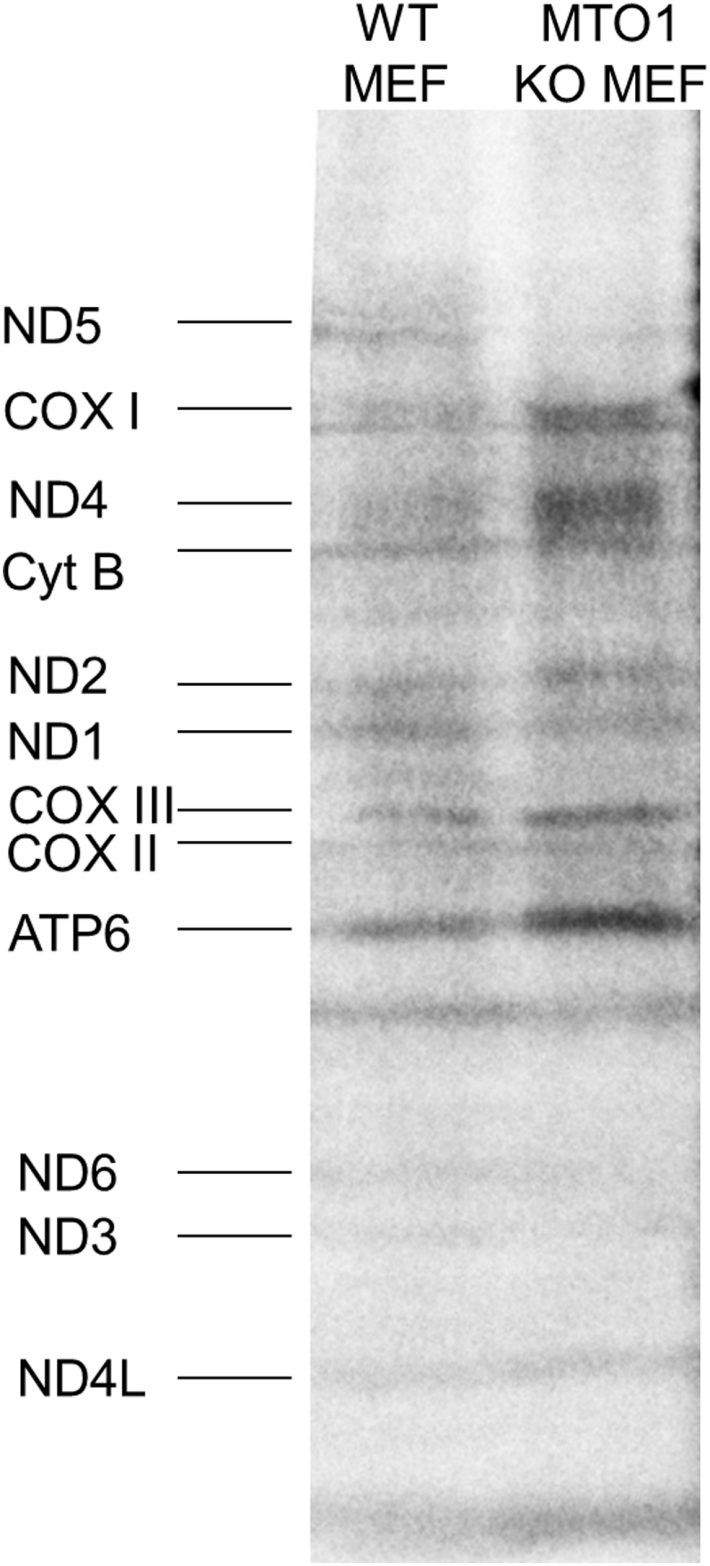
Mouse embryonic fibroblasts experiments. *In vivo* pulse labeling of mitochondrial translation products in WT and *Mto1*−/− showing a clear loss of signal intensity for complex I subunits ND5, ND6 and ND3, while all other mtDNA-encoded proteins showed a trend to increased levels.

To investigate the effects of *Mto1* knockdown on mitochondrial translation, we prepared mouse embryonic fibroblasts (MEFs) from *Mto1* mutant and control mice and performed *in* *vivo* metabolic labeling. We observed an altered pattern in MEFs of *Mto1* animals: there was a clear loss of signal intensity for complex I subunits ND5, ND6 and ND3, while all other mtDNA-encoded proteins showed a trend to increased levels (Fig. 8).

### Mto1−/− mice are more susceptible to paraquat

In a challenge experiment, Mto1 mutant mice and controls were treated with the herbicide paraquat which has been shown to induce oxidative stress [25]. Nine animals of each genotype (14 months old) were injected intraperitoneally with 15 mg/kg in 5-day intervals over 9 weeks. Four out of nine mutants died within this period, while only one out of nine control animals died. Testing the surviving animals after nine weeks of treatment revealed decreased locomotor activity measured as the numbers of squares crossed in the first 30 seconds after transfer to a viewing arena (mutants 2.2±2.4 squares versus control 18.25±6.9 squares; Mann-Whitney Rank Sum Test p<0.001; Figure S6 in S1 File). This was in contrast to the analysis of the untreated cohorts.

## Discussion

While the prevalence and spectrum of human mitochondrial diseases is ever expanding, defects in mitochondrial translation are relatively rarely described. In part, this may be due to the severity of these defects and presumed embryonic lethality in many [25]. Another part of these defects may have escaped diagnosis so far due to lack of awareness, marked clinical heterogeneity and limited diagnostic approaches. The recent advent of exome sequencing markedly facilitates the disclosure of hitherto unassigned disorders, and will likely lead to an increase in the number of mitochondrial translation diseases. A current example is the identification of MTO1 mutations in infantile hypertrophic cardiomyopathy and lactic acidosis [19], [20]. From the three human patients described in the first study, two siblings showed metabolic acidosis, high blood lactate and hypertrophic cardiomyopathy, and died very early (at day 19 and 40, respectively) from sudden bradycardia. Unrelated patient 3 had a much milder course. He developed muscle weakness, failure to thrive, and marked hypertrophic cardiomyopathy in his first months. He had severe metabolic acidosis with high blood lactate. Dichloroacetate treatment resulted in marked improvement of both metabolic acidosis and cardiomyopathy, while plasma lactate remained moderately high. Neurological examination showed mild coordination problems and moderate bilateral optic atrophy [19]. In five additional MTO1 patients, early-onset hypertrophic cardiomyopathy and lactic acidosis associated with respiratory chain deficiency was observed as well [20]. One of the patients was diagnosed with an additional Wolff-Parkinson-White syndrome (MIM: 194200).

The *Mto1* knock-down mouse model presented here mirrors the human phenotype remarkably well. As in patients, the most prominent signs and symptoms were cardiovascular. Both mice and men show a combination of cardiomyopathy and cardiac conduction defects. While the cardiomyopathy is clearly hypertrophic in patients, the findings in mice are more equivocal. Increased left ventricular inner diameters hint, on the one hand, towards dilated cardiomyopathy although fractional shortening was not significantly reduced. On the other hand, increased heart weight despite similar wall thickness suggests hypertrophic cardiomyopathy as seen in most of the patients described. We speculate that the mutation and the subsequent impairment of mitochondrial function leads to heart muscle pathology presenting as dilated or hypertrophic cardiomyopathy depending on additional environmental factors.

Heart from *MTO1* patients has not been investigated so far, but light and electron microscopy of mutant mouse heart tissue revealed myocardial damage with signs of multifocal vacuolization and degeneration as well as ultrastructural changes of mitochondrial morphology.

Bradycardia and cardiac arrhythmias were encountered in both mice and men. Although the exact mechanism remains unclear, we suggest that the reduced heart rate in the Mto1-deficient mice mirrors an underlying pathology of the conduction system. Four out of eight human patients described died in the first year of life. The cause of death was known in three of them, being described as cardio-respiratory arrest in one and as sudden bradycardia in two cases. The mutant mice showed decreased heart rate and marked cardiac arrhythmia, in particular during induction and reversal of anaesthesia. In contrast to the human patients, however, we did not encounter fatal consequences of the cardiac pathology. Recovery from anaesthesia was normal as was the survival in general.

Regarding neurological involvement, psychomotor delay, hypotonia, encephalopathy and seizures have been observed in some Mto1 patients. In the mouse model, testing of muscle strength and coordination testing did not show major disturbances. In the modified Hole Board test, Mto1 mice showed increased horizontal locomotor activity and reduced anxiety-related behaviour, but the significance of this finding remains unclear and it has no obvious correlate in patients. The same is true for the alterations in acoustic startle response that we found in our Mto1 mutant mice.

Hence, the overall pathology seems milder in Mto1-deficient mice as compared to patients. This may, at least in part, be due to the residual expression of full-length *Mto1* transcripts in our knockdown mice which most likely ameliorates the phenotype. In contrast, the human mutations described so far comprise stop mutations or missense mutations of highly conserved positions, e. g. Ala428Thr and Thr411Ile mutations.

On the protein level, there was a clear reduction in full-length mutant MTO1 protein in patient fibroblast mitochondria, whereas an additional truncated MTO1 band was detected [19]. In mice, MTO1 protein levels in fibroblasts were reduced to a lesser extent suggesting that incomplete splicing of the gene trap created a hypomorph allele with residual and fully functional protein levels. Reduced mtDNA content was observed in hearts from mutant mice, a marker generally associated with less mitochondrial number/mass in those tissues. Patient data for mtDNA copy number are not available. An up-regulation of mitochondria and thus mtDNA may be observed in response to oxidative stress [26]. However, mtDNA proliferation and stability is also affected by insufficient mitochondrial translation and transcription. Mutations of genes involved in mtDNA preservation and mitochondrial protein synthesis have been described now on several levels and are modelled in mutant mice. For example, systemic inactivation of the nuclear-encoded mitochondrial transcription factor *Tfam* in mice proved to be embryonic lethal, while heterozygous *Tfam+/−* animals had a partial reduction in the complex I protein level in heart, but not in liver [27]. Reduction of *Tfam* also leads to decline of mtDNA copy number [27], [28]. Yeast strains with defective MTO1 likewise show reduced levels of mtDNA copy number [14] concordant with our finding in *Mto1* mutant mice and also confirming the highly conserved function of MTO1 during evolution. In the hearts analyzed there was a marked reduction especially in Complex I abundance as demonstrated by Western Blot and BNE. In contrast, in skeletal muscle, liver and brain this reduction was much less, in line with the absence of clinical signs of respective dysfunctions. This is also in line with the human data [29].

Complex I consists of 45 polypeptides out of which seven are encoded and transcribed from mtDNA. Complex I deficiency has been shown to cause cardiomyopathies in mice [30], [31]. Chouchani et al. [31] describe mice with selective loss of NDUFS4 in heart showing complex I deficiency as well as severe hypertrophic cardiomyopathy. Quantification of complex I protein in BNE and visualization of complexes with an antibody cocktail revealed clearly reduced amounts of complex I protein in Mto1 mutant mice. The reduction of enzymatic complex I activity, however, was visible but not statistically significant in mouse heart tissue whereas the other complexes measured were not affected. RCC activity data measured in fibroblasts from human patients showed reduced activity especially in complex I and complex IV as well as reduced maximum respiration rates [29]. Mitochondria of Mto1 mutant mice do also show a clear reduction of maximum respiration rates, underscoring the close correlation of human and mouse data. Hill et al. [32] showed that reducing the bioenergetic reserve capacity by 4-hydroxynonenal in cardiomyocytes leads to protein modification and finally cell death. We suggest that the reduced maximal respiration rate detected in human patients and Mto1 mutant mice indicates reduced buffering capacity to face metabolic challenges. We could show an effect of MTO1 deficiency on mitochondrial translation as evident from a metabolic pulse labeling of de novo synthesized mtDNA-encoded proteins. Interestingly, loss of MTO1 seemed to selectively affect complex I subunits such as ND5, ND6 and ND3. This finding is consistent with the observed complex I defect in *Mto1* knockdown animals. In fibroblasts of human patients, no clear differences had been detected [29]. While the molecular basis of MTO1 in the regulation of mammalian mitochondrial translation still remains elusive, our data clearly highlight MTO1 as an important regulator of translation of complex I subunits.

Recent research indicates that mitochondrial dysfunction can result in increased propensity to cardiac arrhythmias (reviewed in [33]) via increased ROS production (e.g. [34], [35]) and impairment of intracellular ion homeostasis and membrane excitability. In this study, we confirm the link between complex I deficiency and liability to cardiac arrhythmias. While a detailed analysis of the molecular mechanisms was beyond the scope of the present study, we found that Mto1 mutants were more susceptible against paraquat-induced oxidative stress (increased mortality, hypoactivity). This suggests that oxidative stress is at least partly involved in the pathogenesis in Mto1 deficiency.

In conclusion, we describe the first mouse model with a defect in mitochondrial translation. Importantly, this *Mto1* knockdown mouse model shows a markedly similar phenotype as compared to the recently described human Mto1 patients. The milder phenotype in the mice (less pronounced cardiomyopathy, normal survival) may be due to residual expression of full-length *Mto1* transcripts in our knockdown mice. Accordingly, there may be much milder affected Mto1 patients than the ones described so far, and it seems worthwhile to include Mto1 mutations in the differential diagnosis of hereditary cardiomyopathies and arrhythmias. Of particular importance is, in our opinion, the fact that cardiac arrhythmias in the mice were only mild to moderate under standard conditions, but became very severe during induction and reversal of anaesthesia. This points towards an increased susceptibility of *Mto1*-deficient mice to additional environmental triggering, e.g. by anaesthetic drugs. While no anaesthesia-related complications were described so far in the few known human Mto1 patients, one translational consequence of this mouse model may be to take precautionary measures in Mto1 patients when they undergo anaesthesia.

## Materials and Methods

### Ethics Statement

Mouse husbandry and all mouse experiments were carried out in accordance with German legal guidelines and were approved by the responsible animal welfare authority, Regierung von Oberbayern, Germany.

### Mice and genotyping

The embryonic stem cell (ES cell) clone G019A03 (Mto1^Gt(G019A03)Wrst^) derived from 129P2/OlaHsd mouse strain was generated using the gene trap vector U3Ceo [39]. The vector insertion site was determined by 50 rapid amplifications of cDNA ends (RACE) and sequencing. ES cells were injected into C57BL/6J host blastocysts to generate chimeras with subsequent germline transmission which were then mated again with C57BL/6J mice. After germline transmission, the heterozygous offspring was interbred to generate homozygous mutants (−/−) and wild-type (+/+) littermate controls. The resulting mixed genetic background is called B6129 S/J). Heterozygous mice were not analyzed. Genotyping was performed on Tail DNA using following primers: 8199F 5′- AGG TAA CAC GGG AAG CTA TC -3′, 8199R 5′- ACC ATA TGA ATC TGA ATC AAC TC -3′ (wildtype) and 8102R 5′- GAT GTC CTG ACC CAA GGC A -3′ (mutant). All mice were maintained on an *ad libitum* diet with a 12-h dark–light cycle.

### Real-time quantitative PCR

Total RNA of 9 control and 9 *Mto1*−/− nitrogen frozen mouse hearts was extracted using TRIZOL Reagent (Invitrogen) and purified with QIAGEN RNeasy MIDI Kit. For cDNA synthesis with Invitrogen SuperScript First-Strand Synthesis System, 2 µg RNA was implemented. Real-time quantitative PCR was performed with the TaqMan Probe-Based Gene Expression Analysis from Applied Biosystems. Probes: TaqManGene Expression Assay for Mouse *Mto1-*spanning exon boundaries but are 5′ to the gene trap integration site (Mm00452767_m1) as well as probes 3′ to the integration site (Mm01171633_g1) and TaqManGene Expression Assay for Mouse *Actinβ* and GAPDH. Data were analysed by StepOne Software v2.0.

### Copy number

DNA was isolated from heart and brain tissue using QIAGEN QIAampDNA Mini and Blood Mini Kit (cat. no.: 51104; Qiagen, Germany) and following the manufacturers’ protocol. The samples were diluted to 1 ng/µl. Copy number variations of mtDNA molecules were defined by quantifying the mtDNA ND1 (NADH dehydrogenase 1) gene relative to the nuclear encoded housekeeping gene Actb using real-time quantitative PCR [24]. Data was evaluated using the 2^∧^–ΔΔCT method.

### Isolation of Mouse Heart Mitochondria

Mice were sacrificed by cervical dislocation; hearts were dissected and homogenized in M2 buffer *(*600 mM Sucrose, 5 mM EDTA, 1 mM PMSF, 50 mM Tris/HCl (pH 7.5)). Unbroken cells and cell nuclei were spun down at 650 g for 7 min at 4°C for three times. Protein concentration of the supernatant was determined using Bradford assay.

### Isolation of Mouse Embryonic Fibroblasts (MEFs)

MEFs were isolated from E13.5 embryos of both genotypes and cultured in DMEM, 10% FKS, 1% sodium pyruvate, 2% Pen/Strep.

### Western Blot

Western Blot using an antibody cocktail binding to subunits of the respiratory chain complexes (MitoProfile Total OXPHOS Rodent WB Antibody Cocktail (ab110413) was performed following the manufacturer’s instructions. For Mto1 specific labelling ab105066 (abcam) was used.

### Determination of specific enzyme activities in isolated mitochondria

Mitochondria were freeze-thawed to make sure that substrates have full accessibility to the mitochondrial inner membrane. The measurement of the specific activity of the individual complexes of the respiratory chain and citrate synthase as mitochondrial activity marker was performed spectrophotometrically, as described [34]. All assays were performed at 37°C.

### Isolation of functional mouse heart mitochondria

Mitochondria were isolated ice cold mitochondrial isolation buffer (MIB) consisting of 300 mM sucrose, 10 mM HEPES, 0.2 mM EDTA, pH 7.2 at 4°C or in addition with 1 mg/ml BSA and pH 7.4 at 4°C (MIB+BSA). Mice were killed by cervical dislocation and the hearts removed and placed in buffer on ice. Fat, vessels, and blood were removed and the hearts quickly minced in a dry dish on ice with scissors to a homogenous rate. With 10 ml of MIB+BSA-buffer the heart sample was homogenized in a Potter-Elvejhem dounce homogenizer with 600 rpm with 10 strokes. Uniform suspension was spun down at 800×g for 10 min at 4°C. Supernatant was then centrifuged at 8000×g for 15 min at 4°C. Pellet was washed with MIB+BSA and MIB subsequently and resuspended in MIB. Protein concentration was measured with the Bradford protocol.

### XF24 assay

Mitochondrial assay solution (MAS) components were: 70 mM sucrose, 220 mM mannitol, 10 mM KH2PO4, 5 mM MgCl2, 2 mM HEPES, 2 mM EGTA, 0.2% (w/v) fatty acid-free BSA, pH 7.2 at 37°C.

Mitochondria were diluted to a concentration of 0,02 µg/µl in MAS buffer containing 10 mM succinate and 2 µM rotenone and seeded in the seahorse plate (50 µl of mitochondria to reach 1 µg/well) on ice. Plate was spun down at 2000×g for 20 minutes at 4°C. After adding pre-warmed 450 µl of MAS buffer containing 10 mM succinate and 1 µM rotenone plate was incubated for 8 minutes at 37°C. Subsequently the following was added to the final concentration of: 4 mM ADP; 2.5 µg/ml oligomycin, 4 µM FCCP and of 20 of 2 µM antimycin A and 1 µM myxothiazol. Data were analyzed with XF software using oxygen consumption rate (OCR pmol/min/well).

### Electrophoresis and quantification of mitochondrial membrane complexes

1-D BNE (blue native electrophoresis) was performed as described [36]. Briefly, 10–500 mg wet weight of specimens from heart were homogenized in sucrose buffer (250 mM sucrose, 20 mM imidazole/HCl, pH 7.0) using a motor-driven tightly fitting 0.5–1 ml glass/Teflon Potter-Elvehjem homogenizer (1,200 rpm, 30 strokes) and centrifuged for 10 min at 22,000×g to obtain enriched mitochondrial membranes. Pellets from 5 mg heart were solubilized in 40 µl solubilization buffer (50 mM NaCl, 50 mM imidazole/HCl, 2 mM 6-aminohexanoic acid, 1 mM EDTA, pH 7.0) and 2 µl 20% dodecyl-β-d-maltoside. Following 5 min incubation on ice and 20 min centrifugation at 22,000 g, protein content of supernatants was measured, equal amounts of protein per sample were supplemented with 1 µl of a 5% Coomassie blue G-250 suspension in 500 mM 6-aminohexanoic acid and loaded onto a 3 to 13% acrylamide gradient gel with a 3% sample gel on top. Following BNE, 1-D gels were stained with Coomassie or by an in gel complex I activity assay (NADH:NBT reductase activity) as described in [37] with some modifications [38]. The Bio-Rad ChemiDoc XRS system was used for densitometric quantification.

### Modified Hole Board and Prepulse Inhibition (PPI) of the Acoustic Startle Reflex (ASR)

The modified Hole Board test was performed as previously described by us [39]. The ASR/PPI protocol was adapted to the specifications of our startle equipment (Med Associates Inc., VT, USA). Background noise (NS = no stimulus) was 65 dB and trial types for ASR included 7 different stimulus intensities (NS, 70, 80, 90, 100, 110, 120 dB). Trial types for PPI included 4 different prepulse intensities (67, 69, 73, 81 dB), each prepulse preceding the startle pulse (110 dB) by 50 msec inter-stimulus interval. ASR/PPI were assessed according to the standardized phenotyping screens developed by the Eumorphia partners [40] available as EMPReSSslim protocols (see www.eumodic.org).

### Neurological examination

Neurological analysis was performed as described [41]. The mice were analyzed for basic neurological functions using a modified SHIRPA protocol, measuring grip strength and rotarod performance for evaluation of motor coordination at different ages.

### Tail-cuff blood pressure measurement

Blood pressure was measured in un-anesthetized mice with a non-invasive tail-cuff method using the MC4000 Blood Pressure Analysis Systems (Hatteras Instruments Inc., Cary, North Carolina, USA). Four animals were restrained on a pre-warmed metal platform in metal boxes. The tails were looped through a tail-cuff and fixed in a notch containing an optical path with a LED light and a photosensor. The blood pulse wave in the tail artery is detected as transformed into an optical pulse signal by measurement of light extinction. Pulse detection, cuff inflation and pressure evaluation are automated by the system software. After five initial inflation runs for habituation, 12 measurement runs are performed for each animal in one session. Runs with movement artifacts are excluded. After one day of training, in which the animals are habituated to the apparatus and protocol, the measurements are performed on four consecutive days between 8∶30 and 11∶30 AM.

### Surface limb ECG

ECG was performed in anesthetized (isoflurane/pressured air inhalation) mice by use of three metal bracelets that are put on the joints of the feet together with electrode gel. The electrodes were positioned on the front-paws and the left hind paw, resulting in the bipolar standard limb leads I, II and III and the augmented unipolar leads AVF, AVR, AVL. ECG is recorded for about seven minutes. Shape analysis of the ECG traces is performed with the software ECG-auto (EMKA technologies, Paris, France). For each animal, intervals and amplitudes are evaluated from five different sets of averaged beats (usually lead II). The parameter Q-T interval is also corrected for the RR interval. In addition, the recordings are screened for arrhythmias, including supraventricular and ventricular extrasystoles and conduction blockages.

### Echocardiography

Left ventricular function was evaluated with transthoracic echocardiography using a Vevo 2100 Imaging System (Visual Sonics) with a 30 MHz probe on conscious animals. Left ventricular parasternal short and long-axis views were obtained in B-mode imaging and left ventricular parasternal short-axis views were obtained in M-mode imaging at the papillary muscle level. The short axis M-mode images were used to measure left ventricular end-diastolic internal diameter (LVEDD), left ventricular end-systolic internal diameter (LVESD), diastolic and systolic septal wall thickness (SWT) and diastolic and systolic posterior wall thickness (PWT) in three consecutive beats. Fractional shortening (FS) was calculated as FS% = [(LVEDD–LVESD)/LVEDD]×100. In addition, heart rate and respiratory rate were calculated by measuring three systolic intervals, respectively three respiratory intervals.

### Pathology examination

Mice received in the laboratory of pathology were sacrificed with CO_2_. The animals were analyzed macroscopically. The body, heart, liver and spleen weight was determined as well as tibia length. All organs were fixed in 4% neutral buffered formalin and embedded in paraffin for histological examination. Four-µm-thick sections of all organs were cut and routinely stained with haematoxylin and eosin (H&E), Congo red, Masson, Periodic acid-Schiff (PAS) and Prussian blue. Two pathologists analyzed each slide independently.

### Transmission Electron Microscopy

Tissue samples were fixed in 2.5% electron microscopy grade glutaraldehyde in 0.1 M sodium cacodylate buffer pH 7.4 (Science Services, Munich, Germany), postfixed in 2% aqueous osmium tetraoxide (Dalton, *1955*), dehydrated in gradual ethanol (30–100%) and propylene oxide, embedded in Epon (Merck, Darmstadt, Germany) and cured for 24 hours at 60°C. Semi-thin sections were cut and stained with toluidine blue. Ultrathin sections of 50 nm were collected onto 200 mesh copper grids, stained with uranyl acetate and lead citrate before examination by transmission electron microscopy (Zeiss Libra 120 Plus, Carl Zeiss NTS GmbH, Oberkochen, Germany). Pictures were acquired using a Slow Scan CCD-camera and iTEM software (Olympus Soft Imaging Solutions, Muenster, Germany).

### Statistical analysis

Data are presented as mean +/− SEM if not stated otherwise. Statistical analysis for two groups was performed using Students t-test if not stated otherwise. Multiple comparisons including sex and genotype were performed using a two way-ANOVA. Differences were considered to be significant at p<0.05.

## Supporting Information

S1 File **Figure S1.** Survival plot. No differences were detected under standard conditions in the survival of mutant mice (n = 259) as compared to wild-type littermates (n = 359). **Figure S2.** CI stain, Quantification of complex I in blue-native gels. A, Protein complexes were solubilized with dodecyl-β-d-maltoside, isolated by blue native electrophoresis and stained by in-gel complex I activity assay. Upper panel: muscle, central panel: liver, lower panel brain. B, Complex I was quantified by densitometry (n = 3). +/+, wild-type mice; −/−, *Mto1* deletion mutants; BHM, bovine heart mitochondria as ladder; error bars indicate standard deviation (SD). **Figure S3.** Muscle: Quantification of OXPHOS complexes in muscle tissue. A, Mitochondrial complexes were solubilized with dodecyl-β-d-maltoside and stained with Coomassie. B, Complexes were quantified by densitometry (n = 3). Assignment of complexes: I, complex I; III, complex III; IV, complex IV; V, complex V. +/+, wild-type mice; −/−, *Mto1* deletion mutants; BHM, bovine heart mitochondria as ladder; error bars indicate standard deviation (SD). **Figure S4.** Liver: Quantification of OXPHOS complexes in liver. A, Mitochondrial complexes were solubilized with dodecyl-β-d-maltoside and stained with Coomassie. B, Complexes were quantified by densitometry (n = 3). Assignment of complexes: I, complex I; III, complex III; IV, complex IV; V, complex V. +/+, wild-type mice; −/−, *Mto1* deletion mutants; BHM, bovine heart mitochondria as ladder; error bars indicate standard deviation (SD). **Figure S5.** Brain: Quantification of OXPHOS complexes in tissue from brain. A, Mitochondrial complexes were solubilized with dodecyl-β-d-maltoside and stained with Coomassie. B, Complexes were quantified by densitometry (n = 3). Assignment of complexes: I, complex I; III, complex III; IV, complex IV; V, complex V. +/+, wild-type mice; −/−, *Mto1* deletion mutants; BHM, bovine heart mitochondria as ladder; error bars indicate standard deviation (SD). **Figure S6.** Brain: locomotor activity after paraquat treatment. Reduced locomotor activity of mutants (n = 6) compared to controls (n = 8) after 9 weeks of paraquat treatment (p<0.001) measured as the number of floor squares crossed after transfer into a viewing arena. **Table S1.** ECG results of an additional cohort of 15 months old mice confirmed the results found in younger animals (cf. Table 1).
(PDF)
