# Supplementary material for: MTO1-Deficient Mouse Model Mirrors the Human Phenotype Showing Complex I Defect and Cardiomyopathy
Source: PLoS One. 2014 Dec 15;9(12):e114918. doi: 10.1371/journal.pone.0114918 (PMC4266617; doi:10.1371/journal.pone.0114918)
Supplement: S1 File — Figure S1. Survival plot. No differences were detected under standard conditions in the survival of mutant mice (n = 259) as compared to wild-type littermates (n = 359). Figure S2. CI stain, Quantification of complex I in blue-native gels. A, Protein complexes were solubilized with dodecyl-β-d-maltoside, isolated by blue native electrophoresis and stained by in-gel complex I activity assay. Upper panel: muscle, central panel: liver, lower panel brain. B, Complex I was quantified by densitometry (n = 3). +/+, wild-type mice; −/−, Mto1 deletion mutants; BHM, bovine heart mitochondria as ladder; error bars indicate standard deviation (SD). Figure S3. Muscle: Quantification of OXPHOS complexes in muscle tissue. A, Mitochondrial complexes were solubilized with dodecyl-β-d-maltoside and stained with Coomassie. B, Complexes were quantified by densitometry (n = 3). Assignment of complexes: I, complex I; III, complex III; IV, complex IV; V, complex V. +/+, wild-type mice; −/−, Mto1 deletion mutants; BHM, bovine heart mitochondria as ladder; error bars indicate standard deviation (SD). Figure S4. Liver: Quantification of OXPHOS complexes in liver. A, Mitochondrial complexes were solubilized with dodecyl-β-d-maltoside and stained with Coomassie. B, Complexes were quantified by densitometry (n = 3). Assignment of complexes: I, complex I; III, complex III; IV, complex IV; V, complex V. +/+, wild-type mice; −/−, Mto1 deletion mutants; BHM, bovine heart mitochondria as ladder; error bars indicate standard deviation (SD). Figure S5. Brain: Quantification of OXPHOS complexes in tissue from brain. A, Mitochondrial complexes were solubilized with dodecyl-β-d-maltoside and stained with Coomassie. B, Complexes were quantified by densitometry (n = 3). Assignment of complexes: I, complex I; III, complex III; IV, complex IV; V, complex V. +/+, wild-type mice; −/−, Mto1 deletion mutants; BHM, bovine heart mitochondria as ladder; error bars indicate standard deviation (SD). Figure S6. Brain: [file pone.0114918.s001.pdf]

Supporting Information Becker et al.

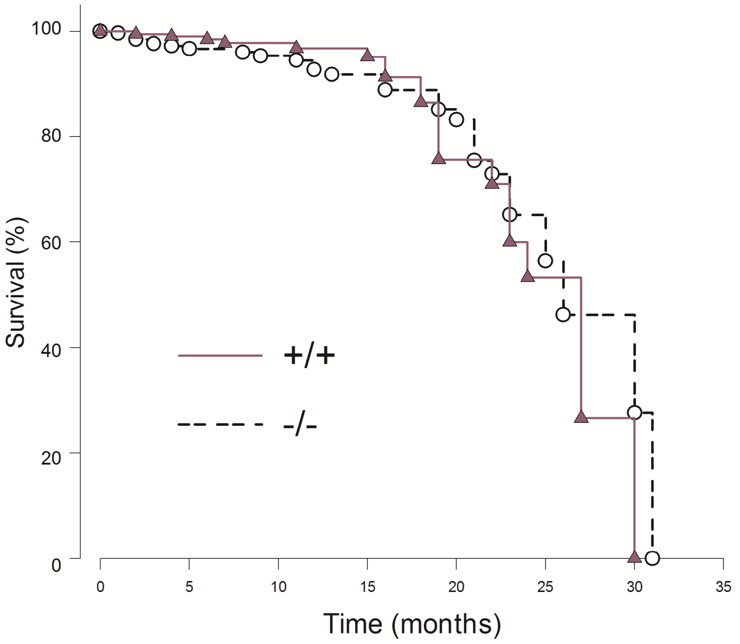

Figure S1:

Survival of mutant mice compared to controls. No differences were detected here under standard conditions (n=359 controls, 259 mutants)

Parameter	Control (A)						Mutant (B)						ANOVA		
	Male			Female			Male			Female			Sex	Genotype	Interact.
	(n =	7	)	(n =	14	)	(n =	21	)	(n =	13	)	*p - value*	*p - value*	*p - value*
**PQ interval [ms]**	37.3	+/-	1.8	37.2	+/-	2.4	39.9	+/-	1.1	40.5	+/-	1.7	**p<0.05**	**n.s.**	**n.s.**
**P-wave duration [ms]**	17.8	+/-	0.7	18.4	+/-	1.0	21.1	+/-	0.4	19.7	+/-	0.8	**n.s.**	**n.s.**	**n.s.**
**QRS-complex duration [ms]**	10.2	+/-	0.2	11.6	+/-	0.9	16.1	+/-	0.6	16.3	+/-	0.4	**n.s.**	**p<0.001**	**n.s.**
**QT interval [ms]**	44.7	+/-	1.6	44.8	+/-	2.4	52.9	1.3	2.8	52.5	+/-	1.5	**n.s.**	**p<0.01**	**n.s.**
**$QT_{corrected}$ [ms]**	39.3	+/-	1.0	39.7	+/-	2.3	40.3	+/-	0.7	39.0	+/-	0.9	**n.s.**	**n.s.**	**n.s.**
**RR interval [ms]**	129.6	+/-	3.1	128.8	+/-	3.5	174.7	+/-	7.2	184.3	+/-	8.6	**n.s.**	**p<0.001**	**n.s.**
**Heart rate [bpm]**	466.1	+/-	10.7	472.1	+/-	12.7	357.6	+/-	14.6	339.1	+/-	13.7	**n.s.**	**p<0.001**	**n.s.**
**JT interval [ms]**	3.8	+/-	0.5	4.9	+/-	0.8	4.6	+/-	0.3	4.5	+/-	0.3	**n.s.**	**n.s.**	**n.s.**
**ST interval [ms]**	34.5	+/-	1.5	33.2	+/-	1.6	36.9	+/-	1.1	36.2	+/-	1.5	**n.s.**	**p<0.05**	**n.s.**
**Q amplitude [mV]**	0.02	+/-	0.00	0.02	+/-	0.00	0.01	+/-	0.00	0.00	+/-	0.00	**n.s.**	**n.s.**	**n.s.**
**R amplitude [mV]**	2.47	+/-	0.33	3.20	+/-	0.30	2.65	+/-	0.18	3.15	+/-	0.20	**n.s.**	**n.s.**	**n.s.**
**S amplitude [mV]**	-1.00	+/-	0.24	-0.67	+/-	0.12	-0.58	+/-	0.09	-0.54	+/-	0.09	**n.s.**	**p<0.05**	**n.s.**
**QRS amplitude [mV]**	3.47	+/-	0.43	3.89	+/-	0.32	3.24	+/-	0.22	3.69	+/-	0.27	**n.s.**	**p<0.05**	**n.s.**
**Arrhythmias**	**SVES**	**VES**	**other**	**SVES**	**VES**	**other**	**SVES**	**VES**	**other**	**SVES**	**VES**	**other**		**Fischer exact**	
**[# of animals]**	0	0	0	0	1	4	2	11	19	0	2	12		**p<0.001**	
**Regular [# of animals]**		7			9			0			1				

Table S1:

ECG results of an additional cohort of 15 months old mice confirmed the results found in younger animals (cf. Table 1)

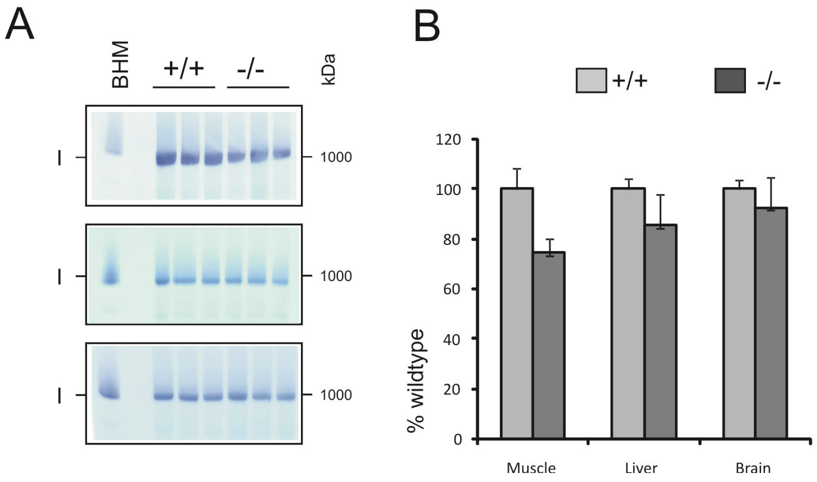

Figure S2 CI stain: Quantification of complex I in blue-native gels.

A, Protein complexes were solubilized with dodecyl-β-d-maltoside, isolated by blue native electrophoresis and stained by in-gel complex I activity assay. Upper panel: muscle, central panel: liver, lower panel brain. **B**, Complex I was quantified by densitometry (n=3). +/+, wildtype mice; -/-, MTO1 deletion mutants; BHM, bovine heart mitochondria as ladder; error bars indicate standard deviation (SD).

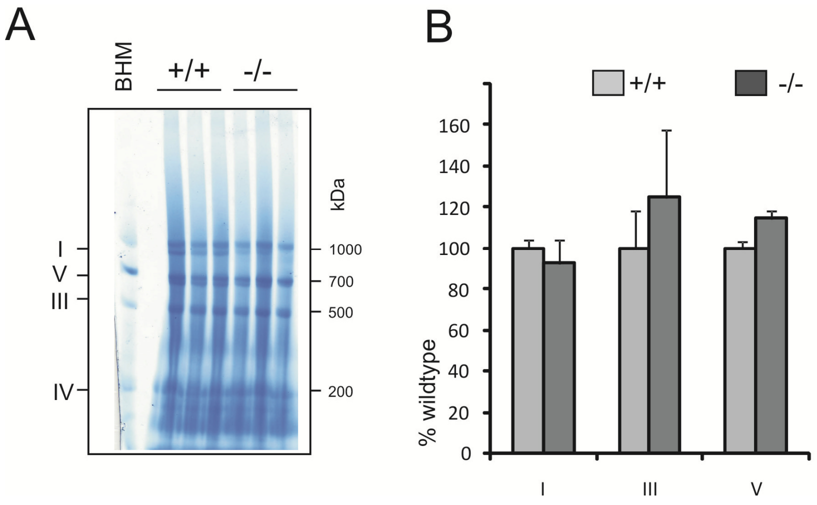

Figure S3 Muscle: Quantification of OXPHOS complexes in muscle tissue.

A, Mitochondial complexes were solubilized with dodecyl-β-d-maltoside and stained with Coomassie. **B**, Complexes were quantified by densitometry (n=3). Assignment of complexes: I, complex I; III, complex III; IV, complex IV; V, complex V. +/+, wildtype mice; -/-, MTO1 deletion mutants; BHM, bovine heart mitochondria as ladder; error bars indicate standard deviation (SD).

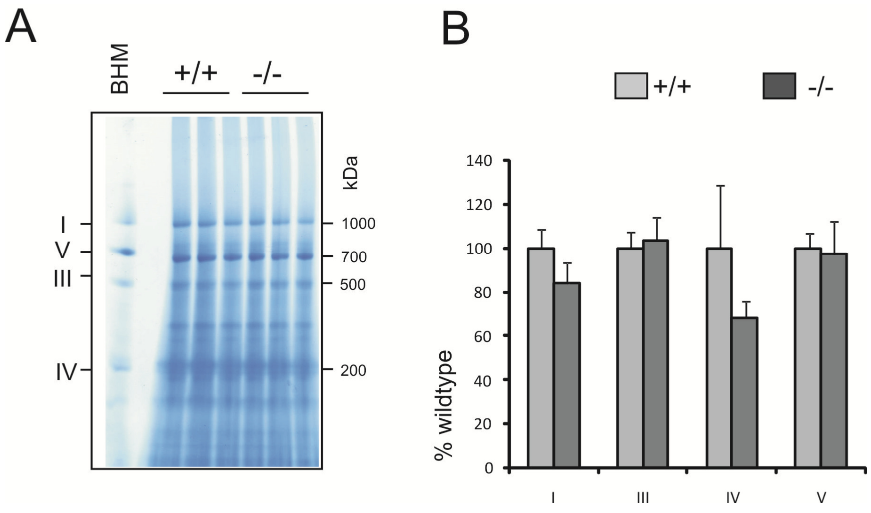

Figure S4 Liver: Quantification of OXPHOS complexes in liver.

A, Mitochondial complexes were solubilized with dodecyl-β-d-maltoside and stained with Coomassie. **B**, Complexes were quantified by densitometry (n=3). Assignment of complexes: I, complex I; III, complex III; IV, complex IV; V, complex V. +/+, wildtype mice; -/-, MTO1 deletion mutants; BHM, bovine heart mitochondria as ladder; error bars indicate standard deviation (SD).

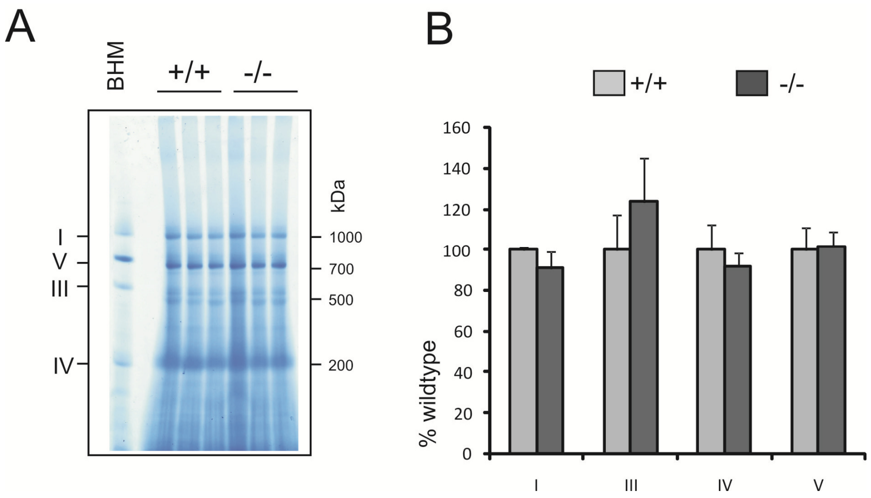

Figure S5 Brain: Quantification of OXPHOS complexes in tissue from brain.

A, Mitochondial complexes were solubilized with dodecyl-β-d-maltoside and stained with Coomassie. **B**, Complexes were quantified by densitometry (n=3). Assignment of complexes: I, complex I; III, complex III; IV, complex IV; V, complex V. +/+, wildtype mice; -/-, MTO1 deletion mutants; BHM, bovine heart mitochondria as ladder; error bars indicate standard deviation (SD).

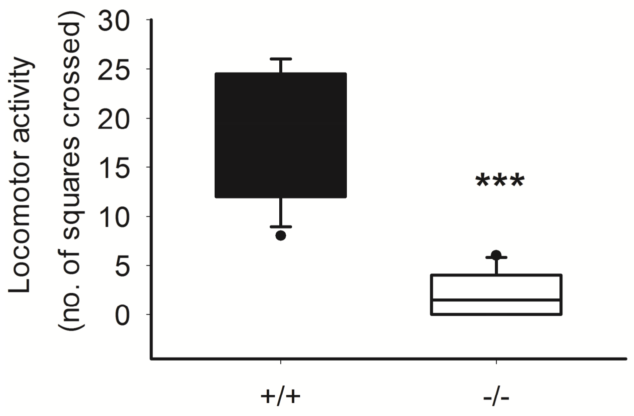

Figure S6 Brain: locomotor activity after paraquat treatment.

Reduced locomotor activity of mutants (n=6) compared to controls (n=8) after 9 weeks of paraquat treatment ($p<0.001$) measured as the number of floor squares crossed after transfer into a viewing arena.
